# Detection of Optogenetic Stimulation in Somatosensory Cortex by Non-Human Primates - Towards Artificial Tactile Sensation

**DOI:** 10.1371/journal.pone.0114529

**Published:** 2014-12-26

**Authors:** Travis May, Ilker Ozden, Benjamin Brush, David Borton, Fabien Wagner, Naubahar Agha, David L. Sheinberg, Arto V. Nurmikko

**Affiliations:** 1 School of engineering, Brown University, Providence, Rhode Island, United States of America; 2 Center for Neuroprosthetics and the Brain Mind Institute, Swiss Federal Institute of Technology (EPFL), Lausanne, Switzerland; 3 Department of Neuroscience, Brown University, Providence, Rhode Island, United States of America; 4 Department of Physics, Brown University, Providence, Rhode Island, United States of America; Emory University, United States of America

## Abstract

Neuroprosthesis research aims to enable communication between the brain and external assistive devices while restoring lost functionality such as occurs from stroke, spinal cord injury or neurodegenerative diseases. In future closed-loop sensorimotor prostheses, one approach is to use neuromodulation as direct stimulus to the brain to compensate for a lost sensory function and help the brain to integrate relevant information for commanding external devices via, e.g. movement intention. Current neuromodulation techniques rely mainly of electrical stimulation. Here we focus specifically on the question of eliciting a biomimetically relevant sense of touch by direct stimulus of the somatosensory cortex by introducing optogenetic techniques as an alternative to electrical stimulation. We demonstrate that light activated opsins can be introduced to target neurons in the somatosensory cortex of non-human primates and be optically activated to create a reliably detected sensation which the animal learns to interpret as a tactile sensation localized within the hand. The accomplishment highlighted here shows how optical stimulation of a relatively small group of mostly excitatory somatosensory neurons in the nonhuman primate brain is sufficient for eliciting a useful sensation from data acquired by simultaneous electrophysiology and from behavioral metrics. In this first report to date on optically neuromodulated behavior in the somatosensory cortex of nonhuman primates we do not yet dissect the details of the sensation the animals exerience or contrast it to those evoked by electrical stimulation, issues of considerable future interest.

## Introduction

Neuroprosthetic research aims to provide tools for direct electronic communication between the brain and external actuators to enable paralyzed people to compensate and partially restore lost motor and sensory functions. Preceded by two decades of research in nonhuman primates with accompanying advances in microelectrode-based neural recording and decoding algorithms, the first clinical trials of motor neuroprostheses have succeeded in recording and decoding activity from hundreds of neurons in the motor cortex, enabling participants with tetraplegia to manipulate a robotic arm with multiple degrees of freedom [1]–[3]. These achievements rely on subjects visual feedback and are thus *open loop* in that no direct stimulus feedback is applied to the brain.

For dexterous manipulation of objects, somatosensation is essential for guiding ongoing movements through interaction with the environment [4]–[6] and for incorporating foreign objects within our body schema [7]. Therefore in a truly closed loop prosthesis, such as operating a robotic hand equipped with haptic sensors, research must strive to not only decode the brains movement intention but also develop methods for writing in sensory information such as tactile cues and proprioceptive information regarding the spatial location of the electronically bidirectional prosthesis [4], [6], [8]. More broadly, ultimate biomimetic sensorimotor neuroprosthetics seek to delivered proxy sensory information by stimulating precisely targeted somatosensory areas of the brain that can be interpreted by the patient as originating from their own or a prosthetic limb.

Recent efforts for delivering direct sensory input to the somatosensory cortex in non-human primates have emphasized the use of electrical stimulation by microelectrodes [9]–[14]. Although electrical stimulation has been shown to generate distinct percepts for sensation [15], the method has limitations for generalization to a high-performance, bidirectional, closed-loop neuroprosthetic. The pathways of current flow and charge delivery in brain tissue are fundamentally challenging to specify and control in electrical stimulation which also generates large noise artifacts that interfere with neural recordings during stimulation periods [11], [16]. In addition, a fundamental question remains unresolved, namely, what and how are specific neural circuit elements biophysically activated or suppressed during electrical stimulation [17]–[19].

Optogenetics offers an alternative to electrical stimulation by providing genetically targeted, cell-type specific activation or inhibition of neural activity by optical means without interfering with neural recordings [20]–[24] and thus compatible with the requirement for simultaneous real-time decoding. A rich literature exists in rodent models highlighting the utility of optogenetic methods for studying neural mechanisms of specific neurological diseases [23], [25]–[28] and information processing at the microcircuit level in the brain [27], [29]–[35]. For example, cell-specific optogenetic stimulation was applied to the somatosensory cortex of a mouse, to induce an apparent illusory touch in a whisker-to-object detection task, revealing mechanisms of somatosensation at a cellular scale [31], [36]. Other optogenetics studies have shown how sensory responses are shaped by the brain states [32] and rhythms [29] which were perturbed by stimulation of certain cell types in the rodent cortex. These basic studies hint at translational possibilities for future sensorimotor neural prosthetics that benefit from selective excitation or inhibition of to manipulate those brain states which gate our perception. The results in rodent models provided impetus to our research in nonhuman primates where the field of optogenetics as a whole is relatively infant despite some promising results within the past 2 years.

Neuromodulation methods have been historically important in nonhuman primate research to study sensory systems at higher levels of functionality such as touch or vision and as a translational step to modeling clinical therapies might perform in scenarios. Related to work in optogenetics, in monkeys we note initial studies which demonstrated that optogenetic stimulation could increase and decrease neural activity using multiple excitatory and inhibitory virus-opsin constructs respectively [37], [38]. One goal of these experiments was to achieve overt behavioral modulation but this has been so far problematic when the stimulus is applied to the motor cortex, in contrast to ready modulation of movements by electrical stimulation. On the other hand, a clear behavioral effect by optogenetic neural activation in the primary visual cortex was reported, which caused monkeys to saccade (gaze) towards the expected visuotopic area based on the optical fiber's stimulating location in the brain. These authors concluded that the monkey most likely experienced a phosphene-like percept upon optical stimulation [39]. Three further studies, all related to the visual system, showed arcuate sulcus activation-delayed saccade timing [40], inhibition in superior colliculus causing predictable saccade deficits [41], and biased visuospatial attention by lateral intraparietal activation using both optogenetic and electrical stimulation [42]. There is considerable current speculation as to the inherent differences between optogenetic and electrical stimulation in non-human primates given the scarcity in the observation of optically modulated behavioral effects. Putative factors include the total number of neurons that express the opsin at the viral injection site and beyond (transfection efficacy), the physical power-dependent illumination volume from the fiber tip light source (versus current spread in charge injection), or perhaps an unspecified preference of electrical stimulation for activating fibers of passage [18]. At the same time, the potential of optogenetics has not yet been explored in the somatosensory cortex to our knowledge. We hypothesized that optogenetics might be effective in this cortical input area where focused and well-defined neural populations can be targeted for significant neuromodulation. Below we demonstrate how optogenetic stimulation generates a robust and reliable sensation with behavioral response features similar to a naturally occurring tactile stimulus.

## Materials and Methods

### Animal Procedures and Ethics Statement

The experiments were performed on two monkeys, S and I (Macaca mulatta, 5.5 kg and 5.9 kg respectively). Primate procedures were approved by the Brown University Institutional Animal Care and Use Committees (IACUC) and performed in accordance with the animal welfare guidelines of the National Institutes of Health.

Animals were kept in Seattle-style stainless steel social environment cages (Suburban Surgical Co., Inc) at the Biomedical Center at Brown University in Providence, Rhode Island, where they remained in the fully enclosed primate room for the duration of the study. The general welfare of the animals was closely monitored to ensure an absolute minimum discomfort. Incorporated into the guidelines was a routine psychological enrichment program, frequent contact with other animals, daily veterinarian supervision and care, and pharmacological amelioration of pain associated with surgeries. Animals were mainly fed a diet of enriched biscuits, fruity prima-treats and assorted fruit as approved by the IACUC. Foraging units, mirrors, music and other toys were provided and consistently rotated to provide enrichment to daily lives of the animals.

**Figure 1:**
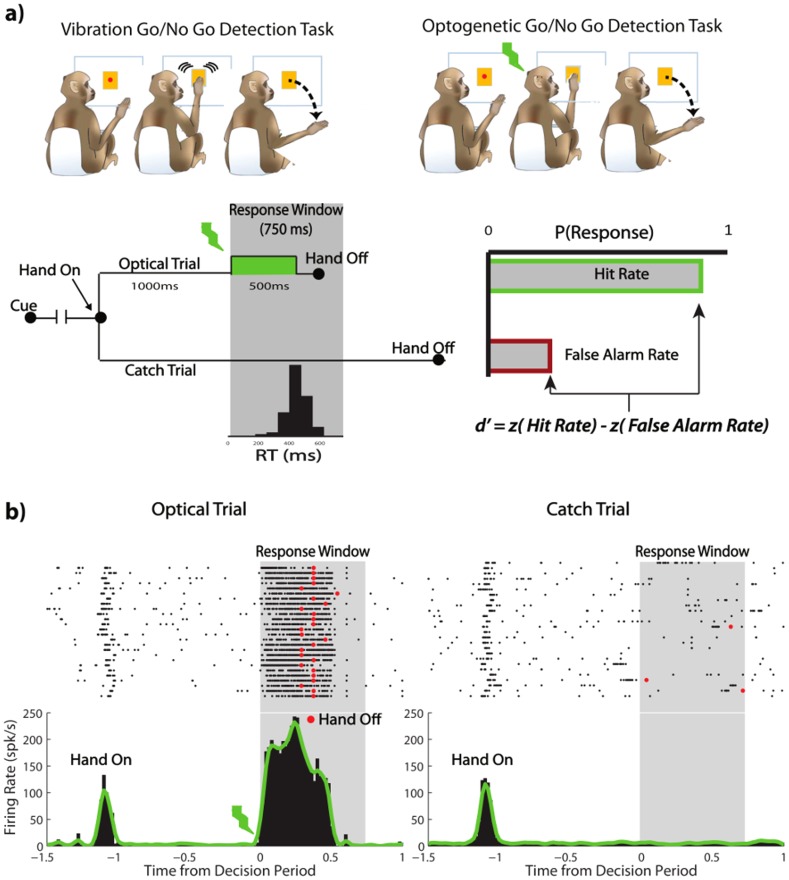
Optogenetic Detection Task. Optogenetic stimulation is consistently detected after an initial learning period. A) Monkeys were first trained on a go/no go vibration detection task. Every trial began with an LED cue to place the hand on the touchpad. The monkeys task was to detect the vibration and respond by quickly removing his hand (<750 ms) to receive a reward. On approximately 50% of trials (Catch Trials), no vibration occurred and the monkey was rewarded for retaining its hand on the touchpad for at least 1.5 seconds. Detection performance was calculated using the proportion of responses on Stim trials (Hit rate) and the proportion of responses on catch trials (False Alarm Rate  =  Chance Performance). After learning the vibration detection task, the monkeys began the optical detection task to detect the optogenetic stimulation delivered directly into the somatosensory cortex (500 ms pulse of continuous stimulation). B) Example raster and peri-stimulus time histogram of multi-unit firing rate during the task. At t = −1 the monkey places its hand on the touchpad causing an increase in firing rate. On Stim Trials at t = 0, the stimulation comes on, increasing the firing rate and the monkey removes its hand if it feels the stimulation (example day with 25 correct trials in a row). On Catch Trials, no stimulation occurs and the monkey retains its hand on the touchpad (Removal during the response window indicates False Alarm).

Each naive animal was first trained on a simple detection task that required an innocuous vibration applied to the finger tips (see Results, Fig. 1). Daily access to fluids was controlled during training and experimental periods to promote behavioral motivation. The animals were engaged in the task for approximately 1–2 hours each day. After learning the required task, each monkey was prepared for surgical procedures that allow access for neural recordings. For surgical procedures, the animals were induced in their home cage with 15 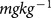 of ketamine then intubated and maintained on isourane (1.4–1.6% MAC) for the remainder of the case. Somatosensory cortex was identified using anatomical landmarks and MRI software (Brainsight, Rogue Research). A cylindrical recording chamber (Crist Instruments) was placed over the identified area and the craniotomy was performed. At the end of one round of experiments, one animal had to be terminated to avoid pain caused by unrelated renal failure. We performed histology by means approved by Brown University IACUC. The brain was sectioned and anti-body stained by outside experts (Neuroscience Associates).

### Viral Injections of Opsin

For opsin expression we used the viral construct AAV5-CamKIIa-C1V1(E122T/E162T)-ts-eYFP (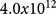 viral-particles per ml) obtained from the Deisseroth Lab (Stanford) and prepared by the University of North Carolina (UNC) vector core. We injected into the hand area of Area 1 of the somatosensory cortex each monkey in a triangular fashion at 5 depths starting 5.5 mm below the cortex and every 1 mm up to 0.5 mm below the cortex (S1 Fig.). Opsin expression in Monkey I was not strong in the first set of injections so we injected a second time. We delivered the second set of injections more lateral to the first set of injections in a triangular fashion but at only 3 depths starting at 3.5 mm below cortex up to 0.5 mm below cortex.

### Neural Recordings and optical stimulation

For simultaneous optical stimulation and recording and minimal tissue damage, we used a coaxial optrode designed for non-human primate use (10 micron tip) [43]. Electrophysiological recordings were performed using a commercial amplifier (AM-Systems), data acquisition card (National Instruments PCI-6024E) and custom code developed in LabVIEW (National Instruments). The first recordings were performed approximately 3 weeks post-injection for each monkey allowing the virus to express. Recordings used in the analysis were all from within 1 mm of the injection sites. We lowered the optrode, occasionally stimulating, until multi-unit activity, monitored through audio feedback, was clearly modulated (Fig. 1). Optical stimulation from a green laser (561 nm, OptoEngine) was delivered at maximum of 2 mW output from the optrode into the tissue. At this point, the receptive field of the single or multi-unit activity was empirically determined by applying pressure to the contralateral hand. The majority of receptive fields for monkey S were on the distal tips of digits 4 and 5. Receptive fields for monkey I were mainly near digits 2 and 3. After receptive fields were mapped, we started the vibration/optical detection trials. For multi-unit data, voltage data was band-passed filtered (300–8000 Hz) and thresholds were determined using 5 times the estimate of the background noise 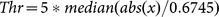. Single units were isolated and artifacts removed using Offline Sorter (Plexon Inc).

### Sensory Task

Each monkey overtly reacted to optical stimulation during passive optical stimulation (i.e. not engaged in the task). They reacted by shaking, rubbing, or staring at the contralateral hand or fingers following the optical stimulation (see S1 Video) they stopped reacting after the initial few optical stimulation trials. We thus sought to define a task to quantify the reliability of the evoked percept. We designed a go/no-go detection task where the animal was first trained to report a physical (mechanical) vibration (see Results). After learning this task he was transferred an optical stimulation detection task (Fig. 1 and S2 Video). We custom designed the vibrating touchpad using cell phone vibrators oscillating near 140 Hz placed behind a capacitive touch sensor to timestamp his reaction time. The setup was controlled using a National Instruments data acquisition system and custom LabVIEW software.

### Behavioral Data Analysis

To quantify the probabilities of the monkey detecting the optical stimulus and reporting this by correctly performing the task, we used standard methods of signal detection theory to compare the number of ‘*GO*’ responses on optical trials—Hit Rate (HR)—with the number of ‘*GO*’ responses on catch trials—False Alarm Rate (FAR) [44]. In Monkey S we performed 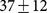 Optical Trials and 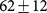 Catch Trials per 100 trial block. In Monkey I we performed 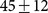 Optical Trials and 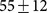 Catch Trials per 100 trial block. 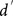 was calculated by taking the difference of the z-scored HR and FAR, and bias towards responding *Go* or *No-Go* was captured using the criterion level, 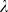: 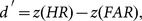


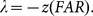



Binomially distributed HR and FAR distributions were used to calculate variances for 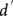 [44]. 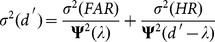
with 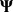 representing the normal probability distribution function. The null hypothesis that 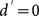 was tested using 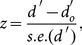
(1)but 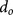 was also be used to test discrimination between two levels of stimulus, e.g. when we compare stimulus duration detection rates. The resulting optogenetic detection learning curves were fit to sigmoid function using 
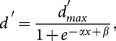
(2)where x is the experimental block number, and 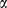 and 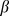 are free parameters. We obtained results for varying the duration of the optical stimulus by fitting the data to an analogous sigmoid function by substituting for x the logarithm of the stimulus duration.

## Results

To answer the question whether optogenetic stimulation in somatosensory cortex could be reliably detected by a NHP, we developed a simple tactile sensory detection task focusing on the hand/digit representation of the primary somatosensory cortex (Area 1). This task paradigm was chosen because it allowed the animals to directly report their detection of the optical stimulus.

We injected two macaque monkeys with a viral vector carrying the channelrhodopsin variant C1V1 targeting Area 1 of the somatosensory cortex, at total of 15 and 21 proximate sites. After allowing maturation of the expression for 3–4 weeks, electrophysiological recordings confirmed that neurons at the injection locations responded to both external tactile stimulation of the contralateral hand as well as to direct cortical optical stimulus. In these measurements, we took advantage of the recently developed coaxial optrode device [43] which enables light delivery and electrical recordings to be made at the same location. As already noted, switching on the optical stimulation unexpectedly caused the monkeys to react by rapidly bringing the hand up to the face for examination, rubbing individual fingers or the palm, or shaking the hand (S1 Video). Although only observational, these reactions suggested that the animals experienced some form of induced tactile sensation localized to the hand/digit areas.

**Figure 2:**
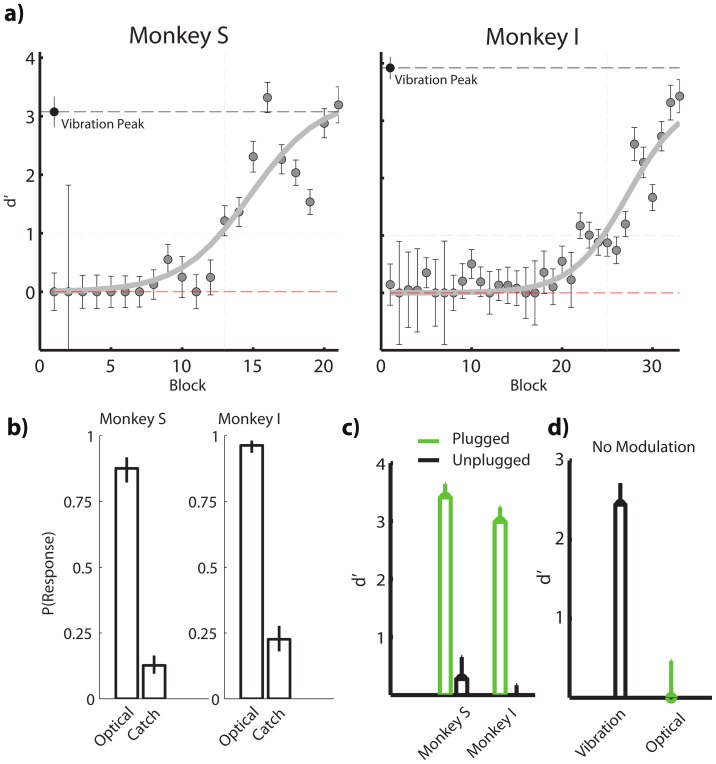
Learning to Detect Optogenetic Stimulation as Proxy for Mechanical Vibration. A) Both monkeys required a learning period when switched to the optical detection task. Every block of trials included 100 trials with approximately 50% stimulation trials and 50% catch trials. Sensitivity index 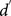 was used to calculate the detectability of the signal in order to compare sessions across blocks where chance performance varies. Peak performance in detecting mechanical vibration is shown as a reference to indicate that both monkeys learned to detect the optical stimulation approaching the performance level of the real physical vibration. Error bars represent the standard error of the mean (grey solid lines represent the sigmoid fit over experimental blocks). B) Summary of the overall detection performance for 500 ms light pulse stimulation for final 6 experimental blocks pooled for each monkey (Monkey S: n = 201 Optical, n = 378 Catch, Monkey I: n = 299 Optical, n = 301 Catch). Error bars are represent standard error of the mean for binomial parameter estimates. Distributions are significantly different by chi-squared (n = 310 and 337, p<<0.001 for both monkeys). C) Control experiment eliminating possible detection of inadvertent external stimuli. Neither neural nor behavioral modulation could be detected with the optical fiber disconnected. D) Control experiment for eliminating light induced heating effects. Stimulation in locations without opsin expression did not modulate neurons nor behavior and could not be detected. Error bars represent standard error of the mean.

In the go/no-go detection task (Fig. 1a), we measured the go/no-go probability using the sensitivity index 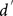, applied from well-established signal detection theory (Methods). On approximately 50% of trials, the stimulus was applied (Stim Trials) and the animal was rewarded for quickly removing its hand (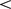
750 ms). On the other 50% of trials, no stimulus occurred (Catch Trials), and the animal was rewarded for retaining its hand on the touchpad for at least 1.5 sec. The response rate during catch trials (False Alarm Rate) for each session defined the chance level while the z-scored difference between response rate on Stim and Catch trials gave us a detectability measure in units of normalized standard deviation. After learning the vibration task, and unbeknownst to the animal, the mechanical vibrations were replaced by a direct optical stimulus of 500 ms duration (2 mW at 561 nm), delivered by the tapered optrode tip into the preselected specific optogenetically transduced targets within Area 1 of somatosensory cortex. We validated the robustness of the C1V1 opsin transduction from histology (S3 Fig.) whereas the spatial accuracy in positioning the coaxial optrode device in vivo was guided by electrophysiological in-situ detection of light modulation of neural activity (Fig. 1b). We observed that the ability of the animals in making the transition from reporting the detection of mechanical vibrations to reporting the percept induced by proxy optical stimulus was not immediate. Performance of Monkey S remained below 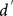 = 1 threshold for the first 1200 trials, while Monkey I took 2500 trials to reach a level of comparable performance (Fig. 2a, S2 Video). The time required for learning suggests that the two types of sensations had some inherent differences which require further investigation. Importantly, however, we found that the animals learned to report percept induced by the proxy light stimulus at performance rates comparable to that delivered by mechanical means. After the initial learning period, the detection rates never fell below chance levels, indicating a consistent effect of the stimulation on behavior. Overall detection rates for the 500 ms long optical stimulation were 87% Hit Rate with a 12% False Alarm Rate for Monkey S, and a 95% Hit Rate with a 23% False Alarm Rate for Monkey I, respectively (Fig. 2b).

Both monkeys responded to mechanical and optical stimuli within 250 to 500 ms indicating that both types of stimuli led to a similar behavioral response in the task (S2 Fig.). To ensure that the animals were responding to the optical stimulation of neurons and not any inadvertent external stimuli, we performed the detection task with the optical fiber disconnected from the coaxial optrode while the device itself remained inserted in the same cortical location. Performance dropped to chance levels when the optic fiber was unplugged (Fig. 2c). Further, to disprove any optically-induced heating effects on the observed results (due to intrinsic tissue light absorption at the stimulation site), we repeated experiments in an area of somatosensory cortex near the injection sites where neural activity was not electrophysiologically modulated by light, and verified that detection rates did not exceed chance levels (Fig. 2d).

**Figure 3:**
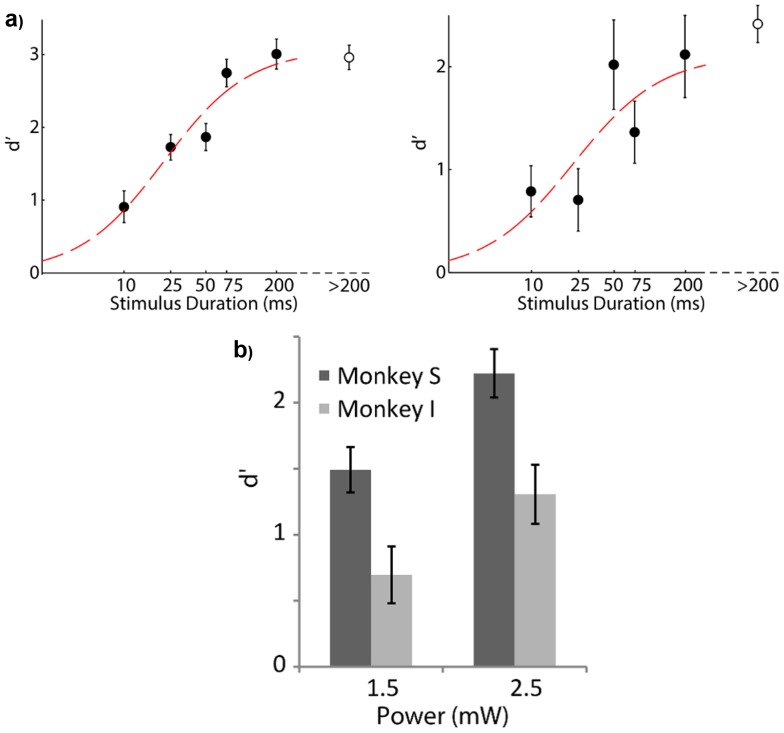
Varying Stimulus Duration and Power Intensity. A) Varying stimulus duration between 10 and 200 ms shows that the optogenetic stimulation is detected with increasing accuracy as stimulus duration increases. Stimulus durations as short as 10 ms can be detected (Monkey S: n = 133 catch trials, and n = [19 30 19 23 19] trials for each duration [10 25 50 75 200] ms; Monkey I: n = 103 catch trials, and n = [25 15 19 21 24]). B) Increasing the power from 1.5 to 2.5 mW causes and increase in the detectability of the optical stimulation in both monkeys. Error bars indicate standard error of the mean. Data are fitted to a sigmoid (dashed lines).

Once the initial learning period for obtaining robust behavioral biomimetic response by optical stimulation was complete, i.e. 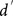
>2.5 for the single 500 ms light pulses (Fig. 2a), we investigated systematic variations in the stimulus duration and laser power during the task (Fig. 3). In the first set of experiments, we kept the laser power constant while using different light pulse durations (between 10–200ms). The detection rate increased with pulse duration but only up to about 100ms (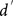 scores were not significantly different for pulse duration of 75 ms and above for Monkey S and within the range from 75–200 ms for Monkey I), indicating that the monkeys only integrated information within this time period (Fig. 3a). Reaction times were independent of pulse duration for both monkeys (p<0.001, t-test with reaction times 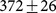 ms and 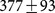 ms for Monkeys S and I, respectively). However, in Monkey I, the reaction times were different between days (days with minimum and maximum reaction times 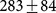 ms and 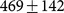 ms, respectively). It thus seems reasonable that the reaction times mainly represent the time at which a decision is made following the percept. Since the reaction times fell within the range of 250 and 500 ms, we pooled all data obtained with pulse durations above 200 ms into one data point in our data analysis (>200 ms). In the second set of experiments, we kept the light pulse duration at 500 ms, but varied the laser power. Here we found that the detection rates were correlated with the amount of (safe) power presented during the decision period. Thus, increasing the power increased detection rate (example in Fig. 3b). As with pulse duration, the reaction times were independent of optical power in a given session.

## Discussion and Conclusions

To our knowledge, our results present the first demonstration that optogenetic stimulation in the somatosensory cortex can be reliably detected by nonhuman primates. Until now intracortical electrical stimulation of this area of the cortex has been the main technique for eliciting distinct sensations for tactile-like percepts within the cortex of nonhuman primates [9]–[14], [45] and humans [15]. Our results may thus offer a starting point for further optogenetics-based sensory neuroprosthetics research, notwithstanding that the parallel efforts to advance research into detailed mechanisms of electrical stimulation and clinical therapies thereof. Although electrical stimulation may fundamentally lack spatial selectivity and specificity, we note that high temporal resolution is still an advantage of electrical stimulation [10]–[12], [45], [46] until the present opsin response time-constants can be lifted which currently limit the effective high-frequency stimulation to approximately 100 Hz depending on the specific opsin.

Our experiment tested whether nonhuman primates could detect optogenetic stimulation of mainly excitatory neurons in the hand/digit areas of the somatosensory cortex. We devised a sensory substitution task whereby we first trained the monkeys on a mechanical detection task and then replaced the vibration with a direct optical stimulus. Our objective was to see whether the optical stimulus might be perceived as a similar sensation to evoke comparable behavioral response. Our monkeys did not immediately report an equivalence between the mechanical and optical stimuli, unlike reported in prior electrical stimulation studies [12], [45], thereby indicating that the optically induced sensation was both distinct and different. We suspect the inability to immediately switch between the mechanical and optical stimuli is partly a result of the present experimental design, in addition from the biophysical differences between optical versus electrical stimulation for eliciting biomimetic tactile sensations. While pragmatic in its simplicity, our mechanical stimulation by a high-frequency vibrating pad challenged the monkeys to maintain focused attention of their hand and digits which increases the odds of detecting a sensory stimulus [11], [36], [46], [47]. Our mechanical task was nonspecific in applying vibration across multiple digits and portions of the palm at the relatively high frequencies (>100 Hz). By contrast, qualitative observation of the monkeys reactions to optical stimulation suggested that this modality of stimulation elicited a well-localized sensation. This contrast could explain the short but delayed transfer of the animals adapting to the proxy optical sensing from their trained mechanical detection.

Historically, it has been known that animals can detect cortical stimulation in almost any area given enough practice [48], but that only stimulation of sensory and motor areas could be immediately detected, as also observed in human patients [15]. Humans appear to detect intraneural stimulation of a single nerve fiber [49] while mice have been reported to detect stimulation of a single somatosensory neuron (above chance levels) [50]. Our results are relevant in this context in that the steep learning curve (Fig. 2) indicates more of a rule-change recognition by the monkeys than reporting on a somewhat different sensation. This recognition would thus guide behavior as opposed to learning curves based on skill acquisition that are evident in cortical areas that do not immediately support detection [51]–[55]. It is noteworthy that as we moved the optrode location across days up to 5 mm laterally in the brain, and the detection rates remained high, indicating that the monkeys could use varying neural populations to drive the same behavior.

While a first report of optogenetic stimulation of the somatosensory cortex for behavioral modulation in nonhuman primates, the outcome from the experiments reported here invite further research for the long term goal of naturalistically replacing/restoring tactile sense of touch of the hand and digits. The strategy of using a targeted optical stimulus needs to be carefully and quantitatively assessed by comparison to the utility of electrical stimulation for which a major challenge remains as to its versatility in substituting for innate tactile experience. There is one report where nonhuman primates were unable distinguish vibrotactile stimuli with electrical stimuli (applied at two distinctly separate frequencies) [12], electrical stimulation in somatosensory cortex has yet to be convincingly shown to be a full surrogate for specific natural tactile stimulus in nonhuman primates [10], [11], [14], [45], [47], [56], [57]. A confounding factor is our present limited state of knowledge about detailed neurophysiological features of natural tactile stimulus, for example, how location, pressure, texture, etc., are represented in the somatosensory cortex [6], [14], [45], [58]. Further, although a spike-count/firing-rate based coding has been suggested to be a major component of sensation [9], [58], [59], evidence suggests that any sensory representation in somatosensory cortex involves coordinated spatial and temporal activity patterns of certain subsets of functional neurons [6], [45], [58], [60], [61]. Accordingly, most electrical stimulation studies to date have focused more on pragmatic demonstrations that stimulation of S1 could recreate a certain functional sensation in a behavioral task, including an indirect interpretation through association, rather than generating a specific biomimetically faithful sensation. We also underscore an important difference between the electrical and optical stimulation, namely the temporal protocols in the two modalities of excitation. Almost universally, electrical stimulation is applied in short(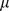
s) but pulses at typical repetition rates on the order of 150 Hz a sets of few hundred millisecond long pulse trains. There are multiple views as to the apparent necessity for such a particular temporal protocol (distinct from the necessity of bipolar charge delivery for electrochemically stability of the electrodes). By striking contrast, which may be due to fundamental differences in the biophysics of stimulation, the optical stimuli applied in this paper consisted of pulses of constant amplitude for the chosen duration (say 500 msec). It is therefore possible that fine tuning the temporal structure of the optical stimulus (including shape of the pulse and deploying trains of short pulses) might lead to a broader repertoire for achieving a more robust proxy tactile sensation. Nonetheless, the utility and simplicity of a constant amplitude excitation strongly suggests that the underlying mechanisms between optical and electrical stimulation have significant biophysical differences as a means to write-in neural information.

In terms of comparing temporal responses between electrical and optical stimulation of the hand/digit area of somatosensory cortex, we emphasize how in our go/no-go task, the detection rates in response to optical stimuli increased steadily with optical pulse durations, reaching a plateau at about 100 ms. Earlier studies with electrical stimulation also indicated similar stimulus durations where peak performance in a sensory detection task was achieved for electrical stimulation durations of (pulse trains) 100 ms or longer [45]. For more complex tasks such as vibrotactile discrimination of mechanical or electrical stimuli, an integration window of at least 250 ms was necessary for successful discrimination [9], [59]. Therefore, the amount of time required for inducing sensory perception by optogenetic stimulation appears similar to electrical stimulation. Another feature of the behavioral responses to optical stimulus was that the reaction times in correct trials did not appear to depend on the optical pulse durations or power within our system time resolution. These results suggest that, under our stimulation conditions, the neural responses at the stimulus onset and the immediately following period of a few tens of milliseconds are important for integrating spikes to help make a decision. Overall, our results may thus represent a first step towards optogenetic neuroprostheses and brain-machine interfaces where the loss of an important sensory modality such as touch could be compensated by cortical stimulus.

## Supporting Information

S1 Fig
**Diagram of Injection Maps.** Left panels show 3D reconstruction of MRI images. Right panels show MRI saggital slices. Both monkeys were injected at three sites perpindicular to the cortex forming a triangle with separation of 1 mm. At each site we injected 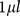 of virus at 3–5 depths. Monkey S was injected at 5 depths from 0.5 to 5.5 mm below cortex. Monkey I was injected on two separate occasions. The first injections were in a triangular fashion with 5 depths. A second set of injections were performed after approximately 3 months because optical modulation and single unit isolation decreased after many optrode penetrations. The second injections were in a triangular fashion with 3 depths 0.5 to 2.5 mm below cortex.(TIF)

S2 Fig
**Comparing Vibration and Optical Reaction Times.** We compared reaction times between the mechanical vibration and optical stimulation detection training paradigms. In the optical detection task, stimulation is delivered directly into the brain, bypassing the neural signal pathway from the finger to the cortex. We predicted that the reaction times for the optical detection task would be shorter by an amount proportional to this conduction latency from finger to cortex which is estimated at 20 ms [62]. We measured the reaction times using a dataset of peak performances for both the vibration and the optical detection tasks (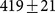 ms vs 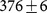 ms for Monkey S, and 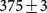 ms vs 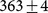 ms for Monkey I 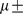 s.e.) and found that the difference in mean reaction times to be 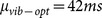 for Monkey S and 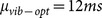 for Monkey I (p = 0.006, p = 0.001, permutation test). Interpretation remains difficult because Monkeys S was not as thoroughly trained on the Vibration Detection task before converting to the optical detection task, thus it is reasonable to believe that the vibration reaction time would be shorter with more practice. In addition, the monkeys are not forced to remove their hand as fast as possible, only fast enough to fall within the 750 ms window, making a direct comparison difficult. Nevertheless, the results seem to suggest the direct intracortical stimulus bypasses the conduction latency from periphery to the brain.(TIF)

S3 Fig **Histology.** Two coronal sections with anti-YFP staining indicating strong C1V1 opsin expression in Monkey S. A) the boxed area shows close-up view of an injection site where many dozens of optrode penetrations were performed over months accumulating finite cortical damage. After many recordings, it became more difficult to record well-defined single units at such sites. B) boxed area shows a second, but less recorded site. Opsin expression was very strong in deeper areas as well, but recordings in this experiment were constrained to the superficial layers. Many injections were performed due to the uncertainty of injection depth and for possible future experiments in area 3b which did not take place. Black scale bars are 1 mm.
(TIF)

S1 Video **Spontaneous Reaction.** Monkey(s) sitting in a chair. When the caption ‘Laser’ appears, a 500 millisecond laser pulse delivers an optical stimulus into the somatosensory cortex, in the location corresponding to the right hand forefingers receptive field. The video shows the animal's spontaneous reaction, whereby the monkey rubs his hands or examines his hands confounded by an unexpected percept. The video frame rate has been slowed down (2X) from real time to help a viewer get a better sense of the monkey's confusion.
(MOV)

S2 Video **Performing Detection Task.** A clip labeled ‘performing task’. Monkey S is performing the go/no-go optogenetic stimulation detection task on a touchpad as follows: On ‘Control Trials’ (Catch Trials), no vibration or optical stimulation takes place - the monkey was trained to report such a ‘null result’ by leaving his hand in contact with the pad>1.5 seconds. On Optical Trials, the laser is delivered directly to somatosensory cortex, with no physical vibration applied. Behaviorally, one sees the monkey reporting the percept by removing his hand quickly (>750 ms).
(MOV)

## References

[1] Collinger JL, Wodlinger B, Downey JE, Wang W, Tyler-Kabara EC, et al (2013) High-performance neuroprosthetic control by an individual with tetraplegia. Lancet 381:557–64. 23253623 10.1016/S0140-6736(12)61816-9 PMC3641862

[2] Hochberg LR, Bacher D, Jarosiewicz B, Masse NY, Simeral JD, et al (2012) Reach and grasp by people with tetraplegia using a neurally controlled robotic arm. Nature 485:372–5. 22596161 10.1038/nature11076 PMC3640850

[3] Hochberg LR, Serruya MD, Friehs GM, Mukand JA, Saleh M, et al (2006) Neuronal ensemble control of prosthetic devices by a human with tetraplegia. Nature 442:164–71. 16838014 10.1038/nature04970

[4] Bensmaia SJ, Miller LE (2014) Restoring sensorimotor function through intracortical interfaces: progress and looming challenges. Nature reviews Neuroscience 15:313–25. 24739786 10.1038/nrn3724 PMC12278825

[5] Johansson RS, Flanagan JR (2009) Coding and use of tactile signals from the fingertips in object manipulation tasks. Nature reviews Neuroscience 10:345–59. 19352402 10.1038/nrn2621

[6] Shaikhouni A, Donoghue JP, Hochberg LR (2013) Somatosensory responses in a human motor cortex. J Neurophysiol 109:2192–204. 23343902 10.1152/jn.00368.2012 PMC3628033

[7] Botvinick M, Cohen J (1998) Rubber hands ‘feel’ touch that eyes see. Nature 391:756. 9486643 10.1038/35784

[8] Weber DJ, Friesen R, Miller LE (2012) Interfacing the somatosensory system to restore touch and proprioception: essential considerations. J Mot Behav 44:403–18. 23237464 10.1080/00222895.2012.735283

[9] Hernandez A, Salinas E, Garcia R, Romo R (1997) Discrimination in the sense of flutter: new psychophysical measurements in monkeys. J Neurosci 17:6391–400. 9236247 10.1523/JNEUROSCI.17-16-06391.1997 PMC6568331

[10] London BM, Jordan LR, Jackson CR, Miller LE (2008) Electrical stimulation of the proprioceptive cortex (area 3a) used to instruct a behaving monkey. IEEE Trans Neural Syst Rehabil Eng 16:32–6. 18303803 10.1109/TNSRE.2007.907544 PMC2586075

[11] O'Doherty JE, Lebedev MA, Li Z, Nicolelis MA (2012) Virtual active touch using randomly patterned intracortical microstimulation. IEEE Trans Neural Syst Rehabil Eng 20:85–93. 22207642 10.1109/TNSRE.2011.2166807 PMC3590844

[12] Romo R, Hernandez A, Zainos A, Salinas E (1998) Somatosensory discrimination based on cortical microstimulation. Nature 392:387–90. 9537321 10.1038/32891

[13] Romo R, Merchant H, Zainos A, Hernandez A (1996) Categorization of somaesthetic stimuli: sensorimotor performance and neuronal activity in primary somatic sensory cortex of awake monkeys. Neuroreport 7:1273–9. 8817548

[14] Tabot GA, Kim SS, Winberry JE, Bensmaia SJ (2014) Restoring tactile and proprioceptive sensation through a brain interface. Neurobiol Dis. 10.1016/j.nbd.2014.08.029 PMC4362964 25201560

[15] Penfield W, Boldrey E (1937) Somatic motor and sensory representation in the cerebral cortex of man as studied by electrical stimulation. Brain 60:389–443.

[16] Rolston JD, Gross RE, Potter SM (2009) A low-cost multielectrode system for data acquisition enabling real-time closed-loop processing with rapid recovery from stimulation artifacts. Front Neuroeng 2:12. 19668698 10.3389/neuro.16.012.2009 PMC2722905

[17] Butovas S, Schwarz C (2003) Spatiotemporal effects of microstimulation in rat neocortex: a parametric study using multielectrode recordings. J Neurophysiol 90:3024–39. 12878710 10.1152/jn.00245.2003

[18] Histed MH, Bonin V, Reid RC (2009) Direct activation of sparse, distributed populations of cortical neurons by electrical microstimulation. Neuron 63:508–22. 19709632 10.1016/j.neuron.2009.07.016 PMC2874753

[19] Tehovnik EJ, Tolias AS, Sultan F, Slocum WM, Logothetis NK (2006) Direct and indirect activation of cortical neurons by electrical microstimulation. J Neurophysiol 96:512–21. 16835359 10.1152/jn.00126.2006

[20] Bernstein JG, Boyden ES (2011) Optogenetic tools for analyzing the neural circuits of behavior. Trends Cogn Sci 15:592–600. 22055387 10.1016/j.tics.2011.10.003 PMC3225502

[21] Deisseroth K (2011) Optogenetics. Nat Methods 8:26–9. 21191368 10.1038/nmeth.f.324 PMC6814250

[22] Packer AM, Roska B, Hausser M (2013) Targeting neurons and photons for optogenetics. Nat Neurosci 16:805–15. 23799473 10.1038/nn.3427 PMC4928704

[23] Tye KM, Deisseroth K (2012) Optogenetic investigation of neural circuits underlying brain disease in animal models. Nat Rev Neurosci 13:251–66. 22430017 10.1038/nrn3171 PMC6682316

[24] Yizhar O, Fenno LE, Prigge M, Schneider F, Davidson TJ, et al (2011) Neocortical excitation/inhibition balance in information processing and social dysfunction. Nature 477:171–8. 21796121 10.1038/nature10360 PMC4155501

[25] Covington HE, Lobo MK, Maze I, Vialou V, Hyman JM, et al (2010) Antidepressant effect of optogenetic stimulation of the medial prefrontal cortex. The Journal of neuroscience: the official journal of the Society for Neuroscience 30:16082–90. 21123555 10.1523/JNEUROSCI.1731-10.2010 PMC3004756

[26] Haubensak W, Kunwar PS, Cai H, Ciocchi S, Wall NR, et al (2010) Genetic dissection of an amygdala microcircuit that gates conditioned fear. Nature 468:270–6. 21068836 10.1038/nature09553 PMC3597095

[27] Kravitz AV, Freeze BS, Parker PR, Kay K, Thwin MT, et al (2010) Regulation of parkinsonian motor behaviours by optogenetic control of basal ganglia circuitry. Nature 466:622–6. 20613723 10.1038/nature09159 PMC3552484

[28] Tye KM, Mirzabekov JJ, Warden MR, Ferenczi EA, Tsai HC, et al (2013) Dopamine neurons modulate neural encoding and expression of depression-related behaviour. Nature 493:537–41. 23235822 10.1038/nature11740 PMC4160519

[29] Cardin JA, Carlen M, Meletis K, Knoblich U, Zhang F, et al (2009) Driving fast-spiking cells induces gamma rhythm and controls sensory responses. Nature 459:663–7. 19396156 10.1038/nature08002 PMC3655711

[30] Guo ZV, Li N, Huber D, Ophir E, Gutnisky D, et al (2014) Flow of cortical activity underlying a tactile decision in mice. Neuron 81:179–94. 24361077 10.1016/j.neuron.2013.10.020 PMC3984938

[31] Huber D, Petreanu L, Ghitani N, Ranade S, Hromadka T, et al (2008) Sparse optical microstimulation in barrel cortex drives learned behaviour in freely moving mice. Nature 451:61–4. 18094685 10.1038/nature06445 PMC3425380

[32] Mateo C, Avermann M, Gentet LJ, Zhang F, Deisseroth K, et al (2011) In vivo optogenetic stimulation of neocortical excitatory neurons drives brain-state-dependent inhibition. Curr Biol 21:1593–602. 21945274 10.1016/j.cub.2011.08.028

[33] Petreanu L, Huber D, Sobczyk A, Svoboda K (2007) Channelrhodopsin-2-assisted circuit mapping of long-range callosal projections. Nat Neurosci 10:663–8. 17435752 10.1038/nn1891

[34] Stamatakis AM, Jennings JH, Ung RL, Blair GA, Weinberg RJ, et al (2013) A unique population of ventral tegmental area neurons inhibits the lateral habenula to promote reward. Neuron 80:1039–53. 24267654 10.1016/j.neuron.2013.08.023 PMC3873746

[35] Warden MR, Selimbeyoglu A, Mirzabekov JJ, Lo M, Thompson KR, et al (2012) A prefrontal cortex-brainstem neuronal projection that controls response to behavioural challenge. Nature 492:428–32. 23160494 10.1038/nature11617 PMC5929119

[36] O'Connor DH, Hires SA, Guo ZV, Li N, Yu J, et al (2013) Neural coding during active somatosensation revealed using illusory touch. Nature neuroscience 16:958–65. 23727820 10.1038/nn.3419 PMC3695000

[37] Diester I, Kaufman MMT, Mogri M, Pashaie R, Goo W, et al (2011) An optogenetic toolbox designed for primates. Nature Neuroscience 14:387–397. 21278729 10.1038/nn.2749 PMC3150193

[38] Han X (2012) Optogenetics in the nonhuman primate, volume 196. Elsevier B.V., 1 edition, 215–33 pp. doi:doi:10.1016/B978-0-444-59426-6.00011-2.. 10.1016/B978-0-444-59426-6.00011-2 PMC3586218 22341328

[39] Jazayeri M, Lindbloom-Brown Z, Horwitz GD (2012) Saccadic eye movements evoked by optogenetic activation of primate v1. Nat Neurosci 15:1368–70. 22941109 10.1038/nn.3210 PMC3458167

[40] Gerits A, Farivar R, Rosen B, Wald L, Boyden E, et al. (2012) Optogenetically Induced Behavioral and Functional Network Changes in Primates. Current Biology: 1–5. 10.1016/j.cub.2012.07.023 PMC3461112 22840516

[41] Ohayon S, Grimaldi P, Schweers N, Tsao DY (2013) Saccade modulation by optical and electrical stimulation in the macaque frontal eye field. J Neurosci 33:16684–97. 24133271 10.1523/JNEUROSCI.2675-13.2013 PMC3797379

[42] Dai J, Brooks DI, Sheinberg DL (2014) Optogenetic and electrical microstimulation systematically bias visuospatial choice in primates. Curr Biol 24:63–9. 24332543 10.1016/j.cub.2013.11.011

[43] Ozden I, Wang J, Lu Y, May T, Lee J, et al. (2013) A coaxial optrode as multifunction write-read probe for optogenetic studies in non-human primates. Journal of neuroscience methods: 1–12. 10.1016/j.jneumeth.2013.06.011 PMC3789534 23867081

[44] Wickens TD (2001) Elementary signal detection theory, volume 1. Oxford University Press, 262 pp.

[45] Tabot Ga, Dammann JF, Berg Ja, Tenore FV, Boback JL, et al. (2013) Restoring the sense of touch with a prosthetic hand through a brain interface. Proceedings of the National Academy of Sciences of the United States of America. 10.1073/pnas.1221113110 PMC3831459 24127595

[46] Venkatraman S, Carmena JM (2011) Active sensing of target location encoded by cortical microstimulation. IEEE transactions on neural systems and rehabilitation engineering: a publication of the IEEE Engineering in Medicine and Biology Society 19:317–24. 10.1109/TNSRE.2011.2117441 21382769

[47] O'Doherty JE, Lebedev MA, Ifft PJ, Zhuang KZ, Shokur S, et al (2011) Active tactile exploration using a brain-machine-brain interface. Nature 479:228–31. 21976021 10.1038/nature10489 PMC3236080

[48] Doty R (1969) Electrical stimulation of the brain in behavioral context. Annual review of psychology. 10.1146/annurev.ps.20.020169.001445 4888623

[49] Ochoa JL (2010) Intraneural microstimulation in humans. Neuroscience Letters 470:162–167. 19818832 10.1016/j.neulet.2009.10.007 PMC3480217

[50] Houweling AR, Brecht M (2008) Behavioural report of single neuron stimulation in somatosensory cortex. Nature 451:65–8. 18094684 10.1038/nature06447

[51] Histed M, Maunsell J (2014) Cortical neural populations can guide behavior by integrating inputs linearly, independent of synchrony. Proceedings of the National … 2013. 10.1073/pnas.1318750111 PMC3890892 24367105

[52] Histed MH, Ni AM, Maunsell JHR (2013) Progress in Neurobiology Insights into cortical mechanisms of behavior from microstimulation experiments. Progress in Neurobiology 103:115–130. 22307059 10.1016/j.pneurobio.2012.01.006 PMC3535686

[53] Ni AM, Maunsell JHR (2010) Microstimulation reveals limits in detecting different signals from a local cortical region. Current biology: CB 20:824–8. 20381351 10.1016/j.cub.2010.02.065 PMC2879058

[54] Murphey DK, Maunsell JHR, Beauchamp MS, Yoshor D (2009) Perceiving electrical stimulation of identified human visual areas. Proceedings of the National Academy of Sciences of the United States of America 106:5389–93. 19276119 10.1073/pnas.0804998106 PMC2664020

[55] Murphey DK, Maunsell JHR (2007) Behavioral detection of electrical microstimulation in different cortical visual areas. Current biology: CB 17:862–7. 17462895 10.1016/j.cub.2007.03.066 PMC2034326

[56] O'Doherty JE, Lebedev MA, Hanson TL, Fitzsimmons NA, Nicolelis MA (2009) A brain-machine interface instructed by direct intracortical microstimulation. Front Integr Neurosci 3:20. 19750199 10.3389/neuro.07.020.2009 PMC2741294

[57] Berg J, Dammann J, Tenore F, Tabot G, Boback J, et al. (2013) Behavioral demonstration of a somatosensory neuroprosthesis. IEEE transactions on neural systems and rehabilitation engineering: a publication of the IEEE Engineering in Medicine and Biology Society. 10.1109/TNSRE.2013.2244616 23475375

[58] Goodwin AW, Wheat HE (2004) Sensory signals in neural populations underlying tactile perception and manipulation. Annu Rev Neurosci 27:53–77. 15217326 10.1146/annurev.neuro.26.041002.131032

[59] Luna R, Hernández A, Brody CD, Romo R (2005) Neural codes for perceptual discrimination in primary somatosensory cortex. Nature neuroscience 8:1210–9. 16056223 10.1038/nn1513

[60] Nicolelis MA, Ghazanfar AA, Stambaugh CR, Oliveira LM, Laubach M, et al (1998) Simultaneous encoding of tactile information by three primate cortical areas. Nat Neurosci 1:621–30. 10196571 10.1038/2855

[61] Ghazanfar AA, Stambaugh CR, Nicolelis MA (2000) Encoding of tactile stimulus location by somatosensory thalamocortical ensembles. J Neurosci 20:3761–75. 10804217 10.1523/JNEUROSCI.20-10-03761.2000 PMC6772666

[62] Nevalainen P, Lauronen L, Pihko E (2014) Development of human somatosensory cortical functions what have we learned from magnetoencephalography: A review. Frontiers in Human Neuroscience 8. 10.3389/fnhum.2014.00158 PMC3955943 24672468

