# Supplementary figures and images for: Detection of Optogenetic Stimulation in Somatosensory Cortex by Non-Human Primates - Towards Artificial Tactile Sensation
Source: PLoS One. 2014 Dec 26;9(12):e114529. doi: 10.1371/journal.pone.0114529 (PMC4277269; doi:10.1371/journal.pone.0114529)

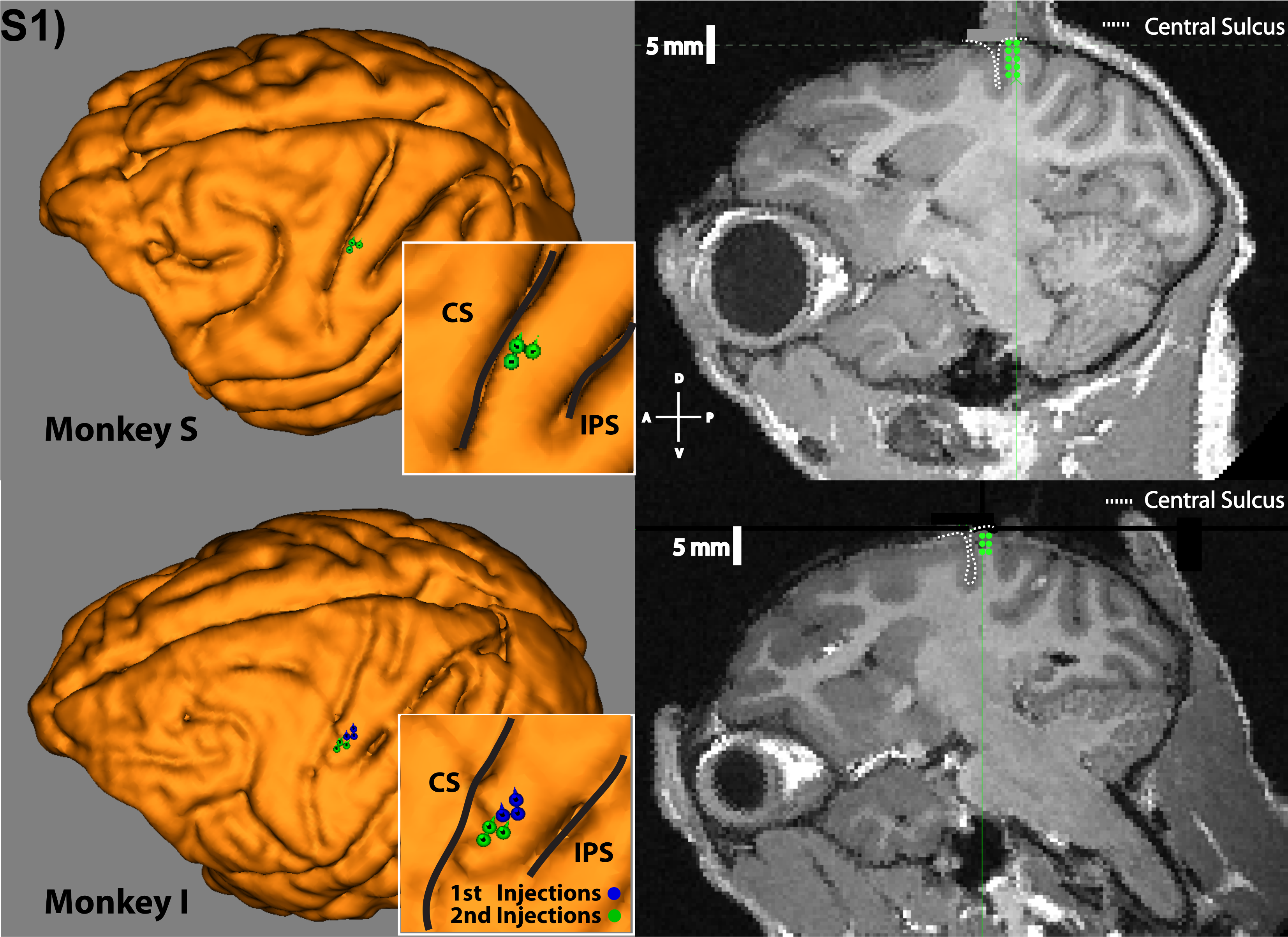

Supplement: S1 Fig — Diagram of Injection Maps. Left panels show 3D reconstruction of MRI images. Right panels show MRI saggital slices. Both monkeys were injected at three sites perpindicular to the cortex forming a triangle with separation of 1 mm. At each site we injected of virus at 3–5 depths. Monkey S was injected at 5 depths from 0.5 to 5.5 mm below cortex. Monkey I was injected on two separate occasions. The first injections were in a triangular fashion with 5 depths. A second set of injections were performed after approximately 3 months because optical modulation and single unit isolation decreased after many optrode penetrations. The second injections were in a triangular fashion with 3 depths 0.5 to 2.5 mm below cortex. (TIF) [file pone.0114529.s001.tif]

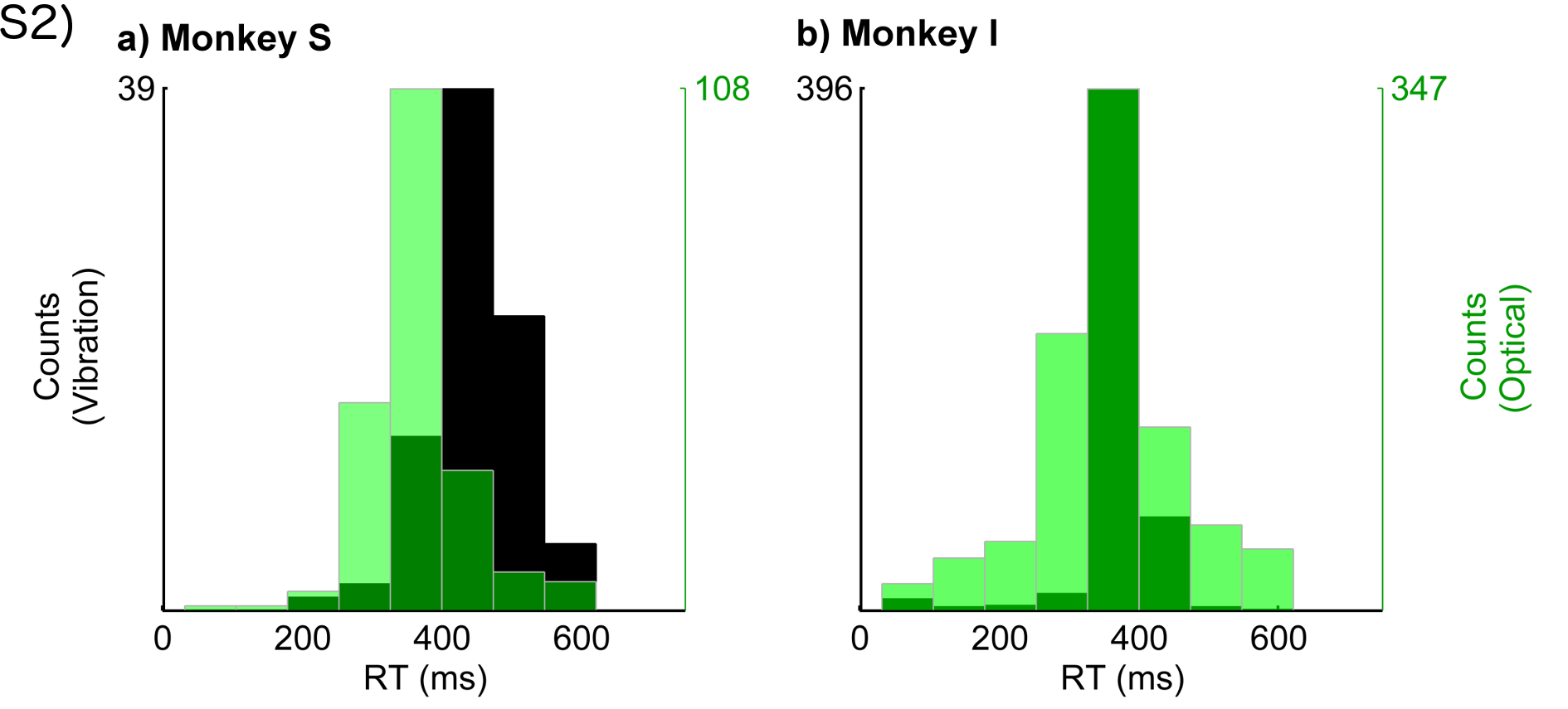

Supplement: S2 Fig — Comparing Vibration and Optical Reaction Times. We compared reaction times between the mechanical vibration and optical stimulation detection training paradigms. In the optical detection task, stimulation is delivered directly into the brain, bypassing the neural signal pathway from the finger to the cortex. We predicted that the reaction times for the optical detection task would be shorter by an amount proportional to this conduction latency from finger to cortex which is estimated at 20 ms [62]. We measured the reaction times using a dataset of peak performances for both the vibration and the optical detection tasks ( ms vs ms for Monkey S, and ms vs ms for Monkey I s.e.) and found that the difference in mean reaction times to be for Monkey S and for Monkey I (p = 0.006, p = 0.001, permutation test). Interpretation remains difficult because Monkeys S was not as thoroughly trained on the Vibration Detection task before converting to the optical detection task, thus it is reasonable to believe that the vibration reaction time would be shorter with more practice. In addition, the monkeys are not forced to remove their hand as fast as possible, only fast enough to fall within the 750 ms window, making a direct comparison difficult. Nevertheless, the results seem to suggest the direct intracortical stimulus bypasses the conduction latency from periphery to the brain. (TIF) [file pone.0114529.s002.tif]
